# CD200 Change Is Involved in Neuronal Death in Gerbil Hippocampal CA1 Field Following Transient Forebrain Ischemia and Postischemic Treatment with Risperidone Displays Neuroprotection without CD200 Change

**DOI:** 10.3390/ijms22031116

**Published:** 2021-01-23

**Authors:** Tae-Kyeong Lee, Myoung Cheol Shin, Ji Hyeon Ahn, Dae Won Kim, Bora Kim, Hyejin Sim, Jae-Chul Lee, Jun Hwi Cho, Joon Ha Park, Young-Myeong Kim, Moo-Ho Won, Choong-Hyun Lee

**Affiliations:** 1 Department of Biomedical Science and Research, Institute for Bioscience and Biotechnology, Hallym University, Chuncheon 24252, Gangwon, Korea; tk-lee@hallym.ac.kr; 2 Department of Emergency Medicine, Institute of Medical Sciences, Kangwon National University Hospital, School of Medicine, Kangwon National University, Chuncheon 24289, Gangwon, Korea; dr10126@naver.com (M.C.S.); cjhemd@kangwon.ac.kr (J.H.C.); 3 Department of Physical Therapy, College of Health Science, Youngsan University, Yangsan 50510, Gyeongnam, Korea; jh-ahn@ysu.ac.kr; 4 Department of Neurobiology, School of Medicine, Kangwon National University, Chuncheon 24341, Gangwon, Korea; nbrkim17@gmail.com (B.K.); janny20@naver.com (H.S.); anajclee@kangwon.ac.kr (J.-C.L.); 5 Department of Biochemistry and Molecular Biology and Research Institute of Oral Sciences, College of Dentistry, Gangnung-Wonju National University, Gangneung 25457, Gangwon, Korea; kimdw@gwnu.ac.kr; 6 Department of Anatomy, College of Korean Medicine, Dongguk University, Gyeongju 38066, Gyeongbuk, Korea; jh-park@dongguk.ac.kr; 7 Department of Molecular and Cellular Biochemistry, School of Medicine, Kangwon National University, Chuncheon 24341, Gangwon, Korea; ymkim@kangwon.ac.kr; 8 Department of Pharmacy, College of Pharmacy, Dankook University, Cheonan 31116, Chungnam, Korea

**Keywords:** CD200, transient ischemia, pyramidal neurons, DND, microglia, GABAergic interneurons, risperidone

## Abstract

It has been reported that CD200 (Cluster of Differentiation 200), expressed in neurons, regulates microglial activation in the central nervous system, and a decrease in CD200 expression causes an increase in microglial activation and neuronal loss. The aim of this study was to investigate time-dependent changes in CD200 expression in the hippocampus proper (CA1, 2, and 3 fields) after transient forebrain ischemia for 5 min in gerbils. In this study, 5-min ischemia evoked neuronal death (loss) of pyramidal neurons in the CA1 field, but not in the CA2/3 fields, at 5 days postischemia. In the sham group, CD200 expression was found in pyramidal neurons of the CA1 field, and the immunoreactivity in the group with ischemia was decreased at 6 h postischemia, dramatically increased at 12 h postischemia, decreased (to level found at 6 h postischemia) at 1 and 2 days postischemia, and significantly increased again at 5 days postischemia. At 5 days postischemia, CD200 immunoreactivity was strongly expressed in microglia and GABAergic neurons. However, in the CA3 field, the change in CD200 immunoreactivity in pyramidal neurons was markedly weaker than that in the CA1 field, showing there was no expression of CD 200 in microglia and GABAergic neurons. In addition, treatment of 10 mg/kg risperidone (an atypical antipsychotic drug) after the ischemia hardly changed CD200 immunoreactivity in the CA1 field, showing that CA1 pyramidal neurons were protected from the ischemic injury. These results indicate that the transient ischemia-induced change in CD200 expression may be associated with specific and selective neuronal death in the hippocampal CA1 field following transient forebrain ischemia.

## 1. Introduction

Transient forebrain ischemia (tFI) is induced by transient deprivation of blood supply to the forebrain and causes a specific and selective neuronal death in the fields vulnerable to tFI. Among the fields, the hippocampus is very vulnerable to tFI [1,2]. In the hippocampus, the pyramidal neurons located in the CA1 field are known to be the most vulnerable after tFI, whereas the pyramidal neurons located in the CA2/3 field are tolerant against tFI [1,3,4]. Especially in the CA1 field, tFI-induced death of the pyramidal neurons occurs a few days (4–5 days) after tFI, and this death is called “delayed neuronal death” (DND) [3]. To explain the mechanisms of the tFI-induced DND, many possible mechanisms, including neuroinflammatory response, have been suggested [5,6,7].

In the normal central nervous system (CNS), microglia participate in neuronal homeostasis, survey the microenvironment, and communicate with neurons [8]. For those, the microglia are influenced by various inhibitory factors, which are produced primarily by neurons [9,10]. Among the signals between neurons and microglia, CD200–CD200R is one of the bidirectional neuron–microglial signals. CD200, a type I membrane glycoprotein, is primarily expressed in neurons in the brain, including hippocampal neurons, and it interacts with its receptor, CD200R, which is primarily expressed in microglia in the brain [11,12,13,14,15]. CD200 in neurons acts as an inhibitory signal, the so-called “do not eat me” signal, to modulate cellular degeneration by microglia [14,16]. Therefore, the interaction between CD200 in neurons and CD200R in microglia maintains a resting state and suppresses phagocytosis by activated microglia in the CNS; therefore, CD200 has been thought to be one of the inhibitors of microglial activity [11,17,18,19].

It has been reported that interactions between neurons and microglia (microglial cells) have been thought to modulate inflammatory responses [20,21]. Some previous studies have demonstrated the changes in CD200 expression in the brain after ischemic damage [7,22,23]. However, the relationship between tFI-induced neuronal damage and CD200 change in parts of the hippocampus (CA1 and CA2/3 fields) after tFI has not been clearly elucidated yet. Therefore, in the present study, we examined the time-dependent changes in CD200 protein expression in the gerbil hippocampus after tFI.

## 2. Results

### 2.1. CD200 Levels

**Figure 1:**
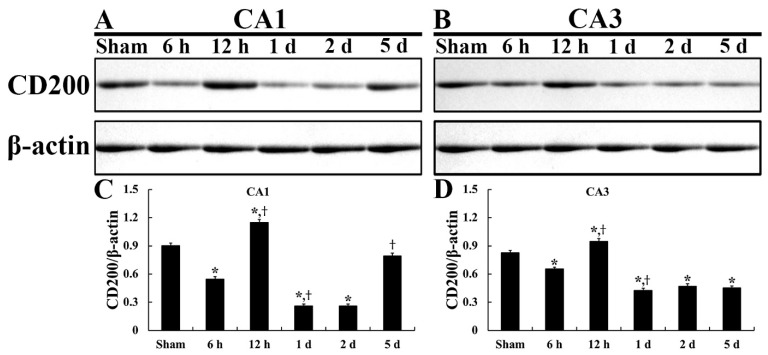
Representative immunoblot of CD200 in the CA1 (**A**) and CA2/3 (**B**) field and their quantified graphs (**C**,**D**). CD200 level is altered in both CA1 and CA3 fields after tFI, showing that the level is decreased from 1 day post-tFI. However, the alteration of the CD200 level after tFI is much more significant in the CA1 field than that in the CA2/3 field. (*n* = 5 at each point in time; * *p* < 0.05, significantly different from the sham group; ^†^ *p* < 0.05, significantly different from the pre-time point group). Bars indicate the means ± SEM.

In the sham group, the fundamental expression level of CD200 was detected in the CA1 and CA3 field (Figure 1A,B). In the ischemia group, CD200 level was remarkably altered in the CA1 field (Figure 1C). CD200 level was reduced by 60.2% at 6 h post-tFI and increased by 127.1% at 12 h post-tFI compared to that in the sham group (Figure 1C). At 1 and 2 days post-tFI, CD200 level was very low (28.7% and 28.7%, respectively, of that in the sham group) (Figure 1C). Five days after tFI, CD200 increased again (87.8% of that in the sham group) (Figure 1C).

In the CA3 field of the ischemia group, the change in CD200 level triggered by tFI was similar with those in the hippocampal CA1 field until 12 h after tFI (Figure 1D) (79.1% at 6 h and 114.4% at 12 h post-tFI compared to those in the sham group). On the other hand, there were no remarkable alterations in the expression level of CD200 in the hippocampal CA3 field 1 to 5 days after tFI, showing that their expression levels of CD200 were significantly weakened (51.4% at 1 d, 56.5% at 2 d, and 54.7% at 5 d post-tFI) compared to those in the sham group (Figure 1D).

### 2.2. DND

**Figure 2:**
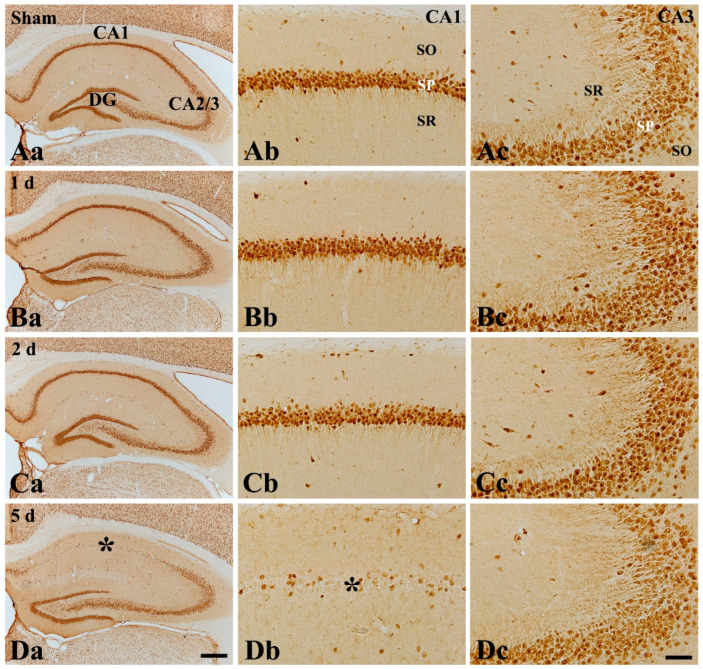
NeuN immunohistochemistry in the hippocampus proper (**Aa**–**Da**), CA1 (**Ab**–**Db**), and CA3 (**Ac**–**Dc**) field of the sham (**Aa**–**Ac**) and ischemia group at 1 day (**Ba**–**Bc**), 2 days (**Ca**–**Cc**), and 5 days (**Da**–**Dc**) after tFI. In the ischemia group, NeuN immunoreactive neurons are not altered at 1 and 2 days after tFI. However, NeuN-immunoreactive neurons are rarely detected in the stratum pyramidale (SP, asterisk) of the CA1 field, and not the CA2/3 fields, at 5 days after tFI. CA—cornu ammonis; SO—stratum oriens; SR—stratum radiatum. Scale bar = 400 µm (**Aa**–**Ca**) and 50 µm (**Ab**–**Db**) and (**Ac**–**Dc**).

Based on the Western blot results, tFI-induced DND in the hippocampus proper was examined in the CA1 and CA2/3 field, respectively, by NeuN immunohistochemistry (Figure 2).

In the sham group, neuronal nuclei-specific protein (NeuN) immunoreactive neurons in the hippocampus proper (CA1–3 fields) were mainly found in the stratum pyramidale (SP) (Figure 2Aa): these NeuN immunoreactive neurons in the SP are called “pyramidal neurons or cells”. In the CA1 field after tFI, the distribution and morphology of NeuN immunoreactive pyramidal neurons at 1 and 2 days post-tFI were not different from those of the sham group (Figure 2Ba,Bb,Ca,Cb). However, NeuN immunoreactive pyramidal neurons were rarely found in the SP at 5 days post-tFI, namely tFI-induced DND occurred in the CA1 pyramidal cells at 5 days post-tFI (Figure 2Da,Db). In the CA2/3 field after tFI, there was no significant alteration of NeuN immunoreactivity until 5 days post-tFI between the sham group and ischemia group (Figure 2Da,Dc). This finding means that DND does not occur in the CA2/3 field, and this finding is consistent with previous results observed by us [4,24,25].

### 2.3. Microgliosis (Microglial Activation)

**Figure 3:**
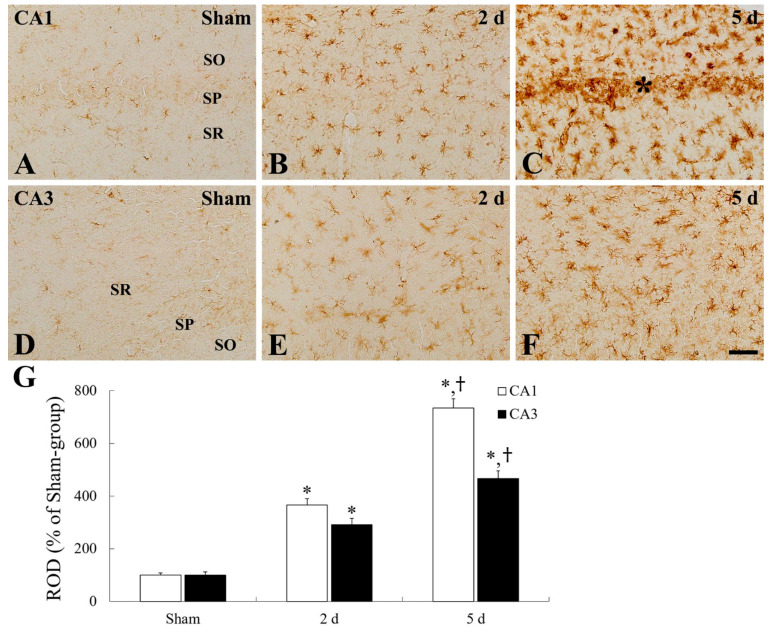
Immunohistochemical staining for Iba-1 in the CA1 (**A**–**C**) and CA2/3 (**D**–**F**) field of the sham group (**A**,**D**) and ischemia group (**B**,**C**,**E**,**F**) at 2 days (**B**,**E**) and 5 days (**A**,**C**,**D**,**F**) after tFI. The activation of Iba-1-immunoreactive microglia increases gradually with time after tFI, showing that tFI-induced Iba-1 immunoreactivity is much higher in the CA1 field than that in the CA3 field. Note that activated microglia are aggregated in the stratum pyramidale (SP, asterisk) at 5 days post-tFI only in the CA1 field. Scale bar = 50 µm. (**G**) Relative optical density (ROD) as percent of Iba-1-immunoreactive structures in the CA1 and CA2/3 field (*n* = 7 at each time, * *p* < 0.05, significantly different from sham group, ^†^ *p* < 0.05, significantly different from pre-time point group). Bars in “G” indicate the means ± SEM.

In the sham group, ionized calcium-binding adapter molecule 1 (Iba-1)-immunoreactive microglia with small cytoplasm and thin processes, as their resting form, were distributed throughout all layers in the CA1–3 fields (Figure 3A,D). In the ischemia group, there was a significant difference in change in Iba-1-immunoreactive microglia between the CA1 and CA2/3 field (Figure 3B,C,E,F). In the CA1 field, Iba-1-immunoreactive microglia were hypertrophied and their Iba-1 immunoreactivity was significantly increased by 366.8% at 2 days and 734.5% at 5 days after tFI, respectively, when compared with those in the sham group (Figure 3B,C,G). In addition, at 5 days after tFI, Iba-1-immunoreactive microglia were highly aggregated in the SP, where tFI-induced DND occurred at this time (Figure 3C). However, in the CA3 field, tFI-induced microglial activation and aggregation of activated microglia were significantly less than those in the hippocampal CA1 field, showing that activated microglia aggregation in the SP was not shown at 5 days after tFI (Figure 3E–G).

### 2.4. CD200 Immunoreactivity

**Figure 4:**
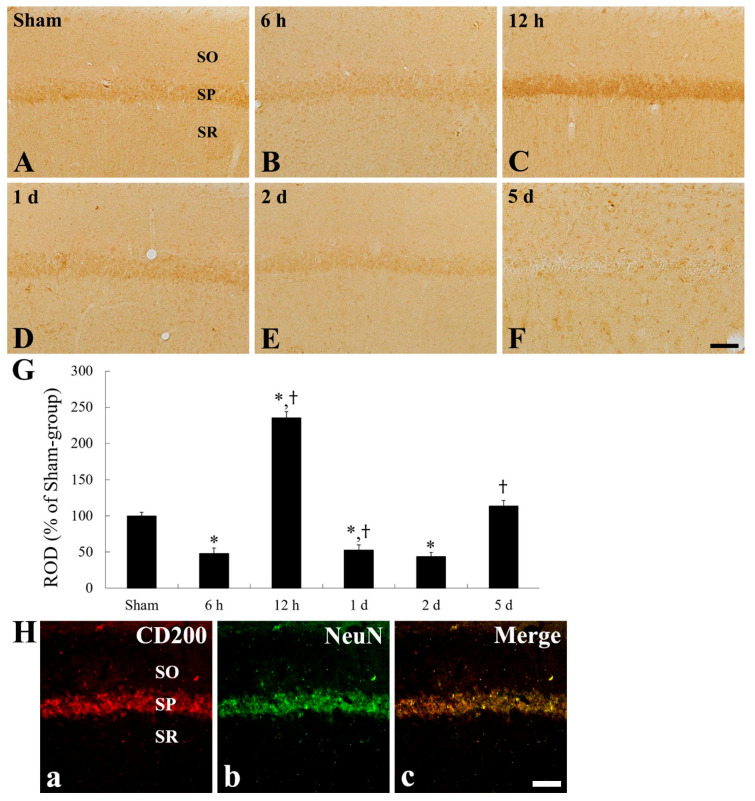
CD200 immunohistochemistry in the CA1 field of the sham group (**A**) and ischemia group (**B**–**F**). CD200 immunoreactivity is significantly decreased in the SP (asterisk) at 6 h after tFI, increased transiently (asterisk) at 12 h after tFI, and decreased again 1 day after tFI. At 5 days after tFI, CD200 immunoreactivity in the SP is hardly shown, but dramatically and newly shown in non-pyramidal cells (arrows) in the SO and SR. Scale bar = 50 µm. (**G**) ROD as % of CD200-immunoreactive structures in the CA1 field (*n* = 7 at each time; * *p* < 0.05, significantly different from sham group; ^†^ *p* < 0.05, significantly different from pre-time point group). (**H**) Double immunofluorescence staining for CD200 (red, (**Ha**)), NeuN (green, (**Hb**)), and merged images (**Hc**) in the CA1 field of the ischemia group at 12 h after tFI. Bars in “G” indicate the means ± SEM.

**Figure 5:**
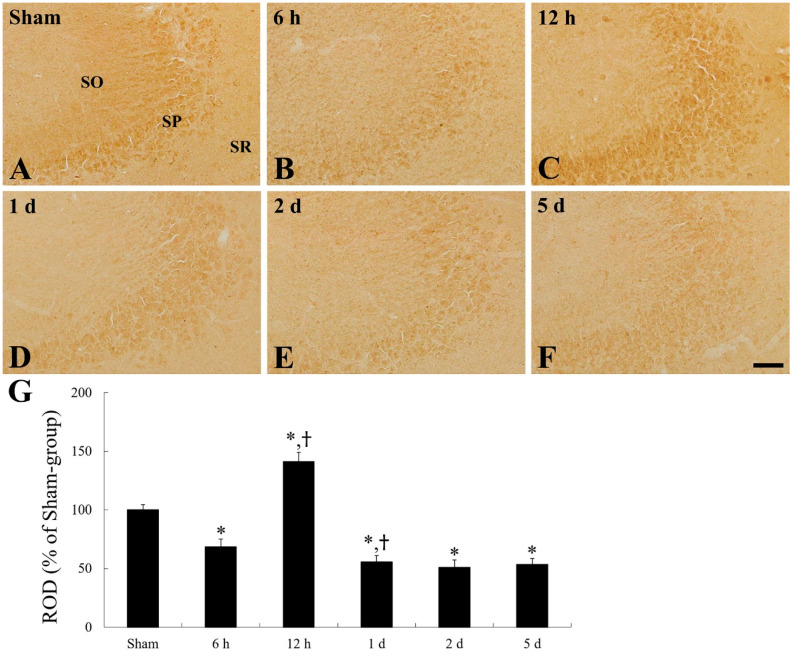
CD200 immunohistochemistry in the CA2/3 field of the sham group (**A**) and ischemia group (**B**–**F**). CD200 immunoreactivity is primarily shown in the SP. CD200 immunoreactivity in the SP is increased at 12 h after tFI; thereafter, CD200 immunoreactivity is decreased and not altered until 5 days after tFI. Scale bar = 50 µm. (**G**) ROD as % of CD200-immunoreactive structures in the hippocampal CA3 field (*n* = 7 at each time; * *p* < 0.05, significantly different from sham group; ^†^ *p* < 0.05, significantly different from pre-time point group). Bars in “G” indicate the means ± SEM.

In the CA1 and CA3 fields of the sham group, moderate CD200 immunoreactivity was primarily detected in pyramidal cells (Figure 4A and Figure 5A), showing that non-pyramidal cells located in the stratum oriens and radiatum showed CD200 immunoreactivity (Figure 4A and Figure 5A).

In the ischemia group, CD200 immunoreactivity was apparently changed in pyramidal cells of the CA1 field (Figure 4B–G). CD200 immunoreactivity was significantly decreased by 47.9% at 6 h after tFI, and increased by 234.7% at 12 h after tFI, compared with that in the sham group (Figure 4B,C,G). Thereafter, CD200 immunoreactivity in the pyramidal cells was decreased with time after tFI (Figure 4D–G). In particular, at 5 days after tFI, CD200 immunoreactivity in the pyramidal neurons was hardly shown, whereas non-pyramidal cells located in the strata oriens and radiatum represented CD200 immunoreactivity (Figure 4F), showing that CD200 immunoreactivity was increased by 113.8% compared with that of the sham group (Figure 4G).

On the other hand, the tFI-induced change pattern of CD200 immunoreactivity in the CA2/3 field was similar to that in the CA1 field (Figure 5B–F); however, the impact of the alteration of the CD200 immunoreactivity in the ischemic CA2/3 field was significantly lower than that in the CA1 field (Figure 5G), showing CD200 immunoreactivity at 5 days after tFI was not altered compared with that at 2 days after tFI (Figure 5F,G).

Meanwhile, to identify the cell types of non-pyramidal cells of newly expressed CD200 in the CA1 field at 5 days after tFI (Figure 4F), we performed double immunofluorescence staining for CD200 with Iba-1 (for microglia) or glutamic acid decarboxylase 65/67 (GABAergic interneurons).

**Figure 6:**
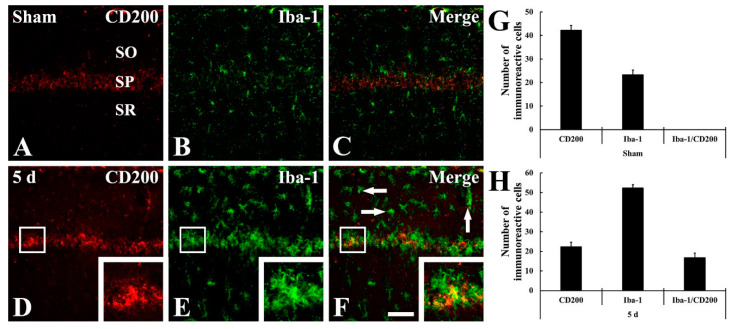
(**A**–**F**) Double immunofluorescence staining for CD200 (red, (**A**,**D**)), Iba-1 (green, (**B**,**E**)), and merged images (**C**,**F**) in the CA1 field of the sham group (**A**–**C**) and ischemia group (**D**–**F**) at 5 days after tFI. CD200 immunoreactivity is not observed in Iba-1-positive microglia of the sham group, whereas CD200 immunoreactivity is shown in Iba-1-positive microglia (arrows) at 5 days after tFI. Scale bar = 50 μm. (**G**,**H**) Analyses of the numbers of CD200 immunoreactive cells, Iba-1 immunoreactive microglia, and Iba-1 immunoreactive microglia among CD200 immunoreactive cells in the sham group (**G**) and ischemia group (**H**).

**Figure 7:**
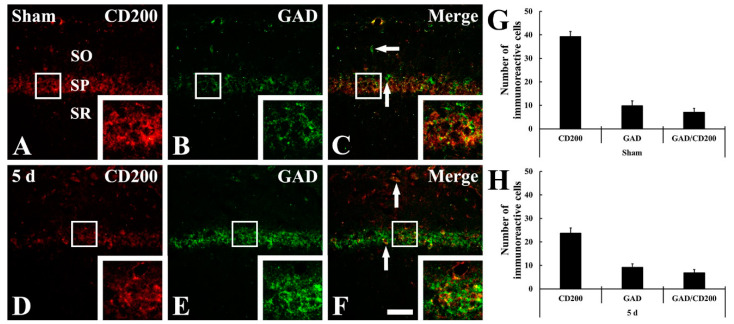
(**A**–**F**) Double immunofluorescence staining for CD200 (red, (**A**,**D**)), GAD65/67 (green, (**B**,**E**)), and merged images (**C**,**F**) in the CA1 field of the sham group (**A**–**C**) and ischemia group (**D**–**F**) groups at 5 days after tFI. GAD65/67-immunoreactive GABAergic interneurons (arrows) show CD200 immunoreactivity in both sham and ischemia groups. Scale bar = 50 μm. (**G**,**H**) Analyses of the numbers of CD200 immunoreactive cells, GAD65/67 immunoreactive GABAergic interneurons, and GAD65/67 immunoreactive GABAergic interneurons among CD200 immunoreactive cells in the sham group (**G**) and ischemia group (**H**).

There was no CD200 immunoreactivity in Iba-1-positive microglia in the sham group (Figure 6A–C). However, at 5 days after tFI, most of the CD200-positive cells were colocalized with microglia (Figure 6D–F). In addition, CD200 immunoreactivity was shown in GAD65/67-immunoreactive GABAergic interneurons in the sham group (Figure 7A–C), and the CD200 immunoreactivity in the GABAergic interneurons was not significantly changed at 5 days after tFI (Figure 7D–F).

### 2.5. Effects of Risperidone (RIS)

**Figure 8:**
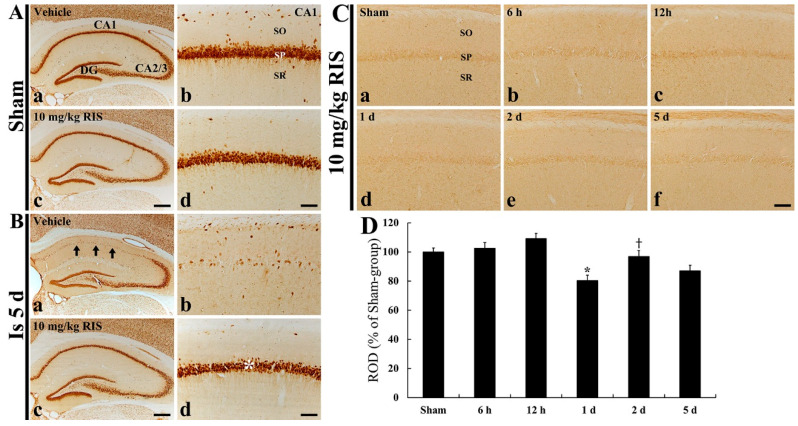
(**A**–**C**) Immunohistochemistry for NeuN (**A**,**B**) and CD200 (**C**) in the hippocampus (**Aa**,**Ac**,**Ba** and **Bc**) and CA1 field (**Ab**,**Ad**,**Bb**,**Bd** and **Ca**–**Cf**) of the sham/vehicle (**Aa**,**Ab** and **Ca**), sham/RIS (**Ac**, **Ad** and **Ca**), ischemia/vehicle (**Ba**,**Bb**), and ischemia/RIS (**Bc**,**Bd** and **Cb**–**Cf**) groups at 5 days after tFI. tNeuN immunoreactive CA1 pyramidal neurons are rarely found in the SP of the CA1 field. However, in the ischemia/RIS group, CA1 pyramidal neurons (asterisk) show strong NeuN immunoreactivity. CD200 immunoreactivity is transiently reduced at 1 day after tFI. Scale bar = 400 µm (**Aa**,**Ac**,**Ba** and **Bc**) and 50 µm (**Ab**,**Ad**,**Bb**,**Bd** and **C**). (**D**) ROD as % of CD200-immunoreactive structures in the CA1 field (*n* = 5 at each time; * *p* < 0.05, significantly different from sham/group; ^†^ *p* < 0.05, significantly different from pre-time point group). Bars indicate the means ± SEM.

Firstly, we observed the effects of RIS on DND in the ischemic CA1 field (Figure 8A,B). In the sham/RIS group, NeuN immunoreactive neurons were not different from those in the sham/vehicle group (Figure 8Aa–Ad). As described above, in the ischemia/vehicle group, the DND of the CA1 pyramidal neurons was shown at 5 days after tFI (Figure 8Ba,Bb). However, in the ischemia/RIS group, NeuN immunoreactive neurons were similar to those in the sham/vehicle group (Figure 8Bc,Bd). This means that RIS treated after tFI protected CA1 pyramidal cells from ischemic injury.

Secondly, we observed the effects of RIS on CD200 immunoreactivity in the ischemic CA1 field (Figure 8C,D). In the sham/RIS group, CD200 immunoreactivity in the CA1 field was not different from that in the sham group as shown in Figure 4A (Figure 8Ca). In the ischemia/RIS group, CD200 immunoreactivity in the CA1 field was not altered until 12 h after tFI (Figure 8Cb,Cc and D). A transiently significant decrease in CD200 immunoreactivity was observed at 1day after tFI compared to that in the sham/RIS group (Figure 8Cd,D). However, CD200 immunoreactivity recovered to the sham level at 2 days after tFI (Figure 8Ce,D), and the immunoreactivity was slightly, not significantly, decreased at 5 days after tFI (Figure 8Cf,D).

## 3. Discussion

In this study, we used only male gerbils as experimental animals. Body structures, hormonal levels, and nature can affect the outcomes of experimental results in both male and female mammals. In female mammals, it is commonly considered difficult to exactly detect their phase of the menstrual cycle; thus, individual female mammals show different levels of sex hormones in plasma from one another. In addition, previous studies have reported sex-dependent differences in ischemic stroke [26,27]. Therefore, we used male Mongolian gerbils in the present study.

In this study, 5-min tFI evoked neuronal death (loss) of pyramidal neurons in the CA1 field, not in the CA2/3 fields, at 5 days after tFI. This finding is consistent with previous studies using gerbils [28,29,30]. Along with the neuronal damage to death, microglial activation was significantly enhanced in the ischemic CA1 field, showing that microglial activation in the ischemic CA2/3 field was apparently lower than that in the CA1 field. This finding is also consistent with previous reports [29,31].

We, in this study, found that CD200 immunoreactivity was principally shown in the pyramidal cells of the CA1–3 fields and that CD200 immunoreactivity was significantly decreased at 6 h after tFI, dramatically increased at 12 h after tFI, and then decreased again from 1 day after tFI in the CA1 field, showing that the tFI-induced change in CD200 immunoreactivity in the CA2/3 field was apparently low compared with that in the CA1 field. In addition, we found that in the ischemic CA1 field, CD200 immunoreactivity was newly expressed in microglia at 5 days post-tFI; CD200 immunoreactivity in the ischemic CA1 field was not expressed in the sham group and until 2 days before tFI. Recently, Yang et al. (2018) showed that CD200 immunoreactivity was found in NeuN-immunoreactive neurons, not in iba-1-immunoreactive microglia, in normal cortical tissue of mice, and that CD200 protein expression was decreased at a lower level from 6 to 48 h after permanent middle cerebral artery occlusion (MCAO) in mice [7]. They stated that the ischemia-induced decrease in CD200 was correlated with neuronal death after MCAO [7]. In addition, it was reported that the expression level of CD200 mRNA in the ischemic core of rat brain following transient focal brain ischemia by MCAO was sharply decreased, reached the minimum amount at 5 days after MCAO, and then increased again up to 14 days after MCAO, showing that CD200 was detected in Iba-1-immunoreactive macrophage-like cells isolated in the ischemic core at 7 days after MCAO [22]. They additionally reported that the marked reduction in CD200 mRNA level in the acute phase after MCAO indicated neuronal degeneration and the increase again after 5 days following MCAO might be due to the infiltration of microglia which expressed both CD200- and Iba-1-immunoreactivity [23].

Interestingly, we found in this study that CD200 immunoreactivity in the CA1 pyramidal neurons markedly decreased at 6 h after tFI, dramatically increased at 12 h after tFI, and thereafter was significantly lower than that in the sham group. This finding is closely related with the microglial activation and DND of CA1 pyramidal neurons following tFI. Namely, the decreased CD200 in the CA1 pyramidal neurons might be involved in the microglial activation, and the increased CD200 might be related with a compensatory mechanism from tFI. Regarding this phenomenon, it was demonstrated that CX3CL1 chemokine, which was normally expressed in pyramidal neurons in the gerbil hippocampal CA1 field, participated in modulating cellular interactions between neurons and microglia after tFI [24]. Namely, CX3CL1 chemokine was decreased in the CA1 pyramidal neurons from 6 h after tFI and dramatically increased at 1 day after tFI [24].

Now, we discuss the new expression of CD200 in Iba-1-positive microglia or macrophages in the CA1 field at 5 days after tFI. It was reported that macrophage-like cells expressing CD200 were accumulated in the ischemic cores of rat brain after transient MCAO [22], suggesting that the CD200 expressed in macrophage-like cells might play a suppressive role in myeloid cell functions in the ischemic core [22]. In addition, Yi et al. (2012) reported that CD200 immunoreactivity was expressed in Iba1-immunoreactive microglia in the hippocampus after kainic acid injection in mice, and CD200 expression was significantly increased after interleukin-4 (IL-4) treatment, not after lipopolysaccharide or lipopolysaccharide with IL-4 treatment in primary microglia, and they suggested that the increase in CD200 expression depended on the alternative activation of microglia.

Additionally, we found in this study that GAD65/67-immunoreactive GABAergic interneurons represented CD200 immunoreactivity at 5 days after tFI, as shown in the hippocampus of the sham group. It is well known that most of GABAergic interneurons, compared with pyramidal neurons in the hippocampal CA1 field, were resistant to cerebral ischemic damage [32,33]. Some underlying mechanisms have been proposed to explain the resistance of GABAergic interneurons in the CA1 field against ischemic damage. For example, it was suggested that increased neurotrophin signaling and the presence of calcium-buffering protein in GABAergic interneurons might protect them from ischemic injury [34,35]. Based on these, we postulate that CD200 expression in GABAergic interneurons does not alter after tFI, which demands why GABAergic interneurons survive in ischemic hippocampus.

In this study, we did not find a dramatic change in CD200 immunoreactivity in pyramidal neurons located in the CA3 field, which were resistant to 5-min tFI. This result is in line with the findings of a previous study reporting that no marked difference in the levels of CD200 mRNA was found in the contralateral cerebral cortex following transient MCAO in rats [23]. Therefore, it can be postulated that the degree of CD200 expression in ischemic neurons is involved in neuronal damage to death following brain insults including tFI. However, more exact roles of CD200 in activated microglia should be investigated in a further study, because we found that CD200 was not expressed in Iba-1-positive microglia in the CA2/3 field, although the Iba-1-positive microglia were hypertrophied (activated) after tFI.

Finally, we investigated CD200 immunoreactivity in the ischemic CA1 field after postischemic treatment with 10 mg/kg RIS, because we recently demonstrated that therapeutic treatment with 10 mg/kg RIS after 5-min tFI in gerbils displayed excellent neuroprotective effects in a gerbil model of tFI and a rat model of MCAO-induced transient focal cerebral ischemia [36,37]. We found that posttreatment with RIS strongly protected pyramidal neurons in the ischemic CA1 field: this finding is consistent with the previous finding. For CD200 immunoreactivity, we found that under neuroprotective conditions, CD200 immunoreactivity was not changed in the ischemic CA1 pyramidal neurons. This finding means that tFI-induced DND is not triggered when RIS is treated after tFI, showing that CD200 expression is not affected by tFI. Yang et al. (2018) reported that intracerebroventricular injection with recombinant CD200 protein attenuated microglial activation and the expression of pro-inflammatory cytokines (TNF-α, IL-1β, and IL-10) following MCAO in rats [7].

In summary, both CD200 expression level and immunoreactivity in the ischemic hippocampal CA1 field of gerbil were significantly changed following tFI, showing that CD200 was newly expressed in activated microglia in the CA1 field at 5 days post-tFI, at which time the DND of the CA pyramidal neurons died. These results indicate that tFI-induced change in CD200 expression may be associated with the selective DND of pyramidal neurons located in the hippocampal CA1 field following 5-min tFI. The current results also displayed that neuroprotection against tFI accomplished by post-treatment with 10 mg/kg risperidone provided the maintenance of CD200 immunoreactivity in the hippocampal CA1 field following tFI. In this regard, we suggest that targeting CD200 can be useful as a therapeutic strategy under ischemic conditions.

## 4. Materials and Methods

### 4.1. Experimental Animals

A total of 130 male gerbils were used at 6-months-old (body weight, 80 ± 5 g), which were provided by the Experimental Animal Center of Kangwon National University (Chuncheon, Kangwon, Korea). They were housed in a conventional state (room temperature, 23 ± 0.5 °C; humidity, 55 ± 5%; 12:12 light/dark cycle) with freely accessible pellet feed and water. The experimental protocol was approved (approval no., KW-200113-1; approval date, 7 February 2020) by the Institutional Animal Care and Use Committee (IACUC) of Kangwon National University.

### 4.2. Induction of tFI

**Figure 9:**
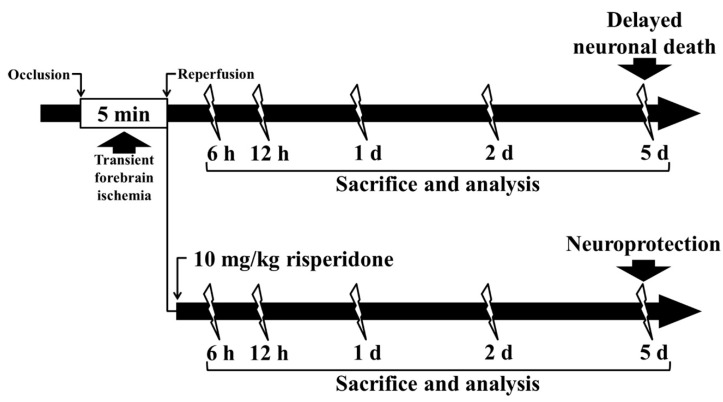
Experimental timeline. The gerbils underwent 5-min tFI. They were sacrificed at 6 h, 12 h, 1 d, 2 d, and 5 d after tFI, and their hippocampi were analyzed using immunohistochemistry and Western blot analysis. The gerbils post-treated with 10 mg/kg RIS were sacrificed at 6 h, 12 h, 1 d, 2 d, and 5 d, and their hippocampi were analyzed by immunohistochemical staining.

As previously described in our published papers [4,24,25], the surgical procedure of tFI in the gerbils was performed. Briefly, the gerbils were anesthetized with a mixture of 2.5% isoflurane (Baxtor, Deerfield, IL, USA) in 67% nitrous oxide and 33% oxygen. Under anesthesia, both common carotid arteries located at the neck were isolated and ligated for 5 min with aneurysm clips (Figure 9). Body temperature was checked with a rectal temperature probe and controlled at normothermic conditions (37 ± 0.5 °C) with a thermometric blanket during and after tFI. The sham gerbils received the same tFI operation without the occlusion of the carotid arteries.

### 4.3. Western Blotting

Western blot analysis was conducted according to previous studies [38,39]. In short, five gerbils at each point in time per group were profoundly anesthetized by intraperitoneal administration of pentobarbital sodium (90 mg/kg) (JW pharm. Co. Ltd., Seoul, Korea). The gerbils were decapitated, and their brains were harvested. These brains were homogenized with a buffer for homogenization (50 mM PBS; pH 7.4) containing 0.1 mM EGTA (pH 8.0), 0.2% Nonidet P-40, 10 mM EDTA (pH 8.0), 15 mM sodium pyrophosphate, 100 mM β-glycerophosphate, 50 mM NaF, 150 mM NaCl, 2 mM sodium orthovanadate, 1 mM PMSF, and 1 mM DTT. The homogenized brain tissues were centrifuged, and the supernatants were taken to evaluate protein levels with a Micro BCA assay kit (Thermo Fisher Scientific Inc., Waltham, MA, USA) with bovine serum albumin (Pierce Chemical, Rockford, IL, USA) as a standard. Aliquots including total protein (20 μg) were boiled in loading buffer containing 150 mM Tris (pH 6.8), 3 mM DTT, 6% SDS, 0.3% bromophenol blue, and 30% glycerol. The samples were separated by SDS-PAGE (10%) and the gels were subsequently transferred to nitrocellulose membranes (Pall Co., East Hills, NY, USA) at 350 mA and 4 °C for 90 min. To block non-specific staining, the membranes were incubated with 5% defatted milk at room temperature for 1 h. The membrane, thereafter, was immunoreacted with each primary antibody: goat anti-CD200 (diluted 1:1000) (R&D systems, Minneapolis, MN, USA) and rabbit anti-β-actin (diluted 1:2000) (Sigma-Aldrich, St. Louis, MO, USA) at 4 °C for 9 h. In addition, the membrane was incubated with each HRP-conjugated secondary antibody: rabbit anti-goat IgG (diluted 1:2000) (R&D systems, MN, USA) and donkey anti-rabbit IgG (diluted 1:5000) (Santa Cruz Biotechnology, Santa Cruz, CA, USA) for 1 h. Finally, to enhance visualization, a luminol-based chemiluminescence kit (Pierce; Thermo Fisher Scientific Inc., Waltham, MA, USA) was used.

The Western blots were analyzed as previously described [40]. Briefly, each band was scanned and densitometrically analyzed for quantification using the Scion Image software (Scion Crop., Frederick, MD, USA). Each protein level was normalized via the corresponding level of β-actin. To avoid bias, all measurements in the present study were performed by two observers for each experiment.

### 4.4. Preparation of Brain Sections

Brain sections containing the hippocampus were prepared as previously described in our studies [4,24,25]. In brief, the gerbils in the sham (*n* = 5 at each point in time) and tFI (*n* = 7 at each point in time) group were deeply anesthetized for euthanasia with 70 mg/kg of pentobarbital sodium at 6 h, 12 h, 24 h, 2 days, and 5 days after sham and tFI operation (Figure 9). Under anesthesia, the gerbils were rinsed transcardially with saline, and their brains were perfused to be fixed with 4% paraformaldehyde solution (in 0.1 M PB, pH 7.4). The brains were removed from the skulls and fixed again in the same fixative for 6 h. For cutting the brains, they were infiltrated with 30% sucrose solution (in 0.1 M PB, pH 7.4) to avoid tissue damage from freezing. Finally, the brain tissues were coronally sectioned to 30-µm thickness in a cryostat.

### 4.5. Immunohistochemistry for NeuN, Iba-1, and CD200

According to previously published methods [4,24,25] with modification, immunohistochemical stainings for NeuN to detect neurons, Iba-1 to detect microglia, and CD200 were performed by streptavidin–biotin–peroxidase method. In brief, the sections were treated with 0.3% hydrogen peroxide (H_2_O_2_) for 20 min at room temperature to block endogenous peroxidase activity, and 5% normal horse serum was treated for 30 min at room temperature to block unspecific proteins in the tissues. Then, tissues were incubated with mouse anti-NeuN (diluted 1:1,100, Chemicon, Temecula, CA, USA), rabbit anti-Iba-1 (diluted 1:900, Wako Chemicals, Japan), and goat anti-CD200 (diluted 1:100, R&D systems, MN, USA), respectively, for 10 h at 4 °C. The sections were then rinsed, incubated with biotinylated horse anti-mouse or anti-rabbit or anti-goat IgG (Vector Laboratories, Burlingame, CA, USA) for 2 h at room temperature, and continuously reacted with Elite avidin–biotin enzyme complex (ABC) (Vector Laboratories Inc., Burlingame, CA, USA) for 1 h. The visualization of each immunoreaction was achieved with solution of 3, 3′-diaminobenzidine (DAB) (Vector Laboratories Inc., Burlingame, CA, USA). In order to establish the specificity of each immunoreaction, each negative control test was performed using pre-immune serum instead of each primary antibody. We confirmed no immunoreactivity in each immunostained tissue.

Images of each immunoreactive structure were captured in the CA1 and CA2/3 fields for quantitative analysis. We used a BX53 microscope from Olympus (Tokyo, Japan) equipped with a DP72 digital camera (Olympus, Tokyo, Japan) and used cellSens Standard (Olympus, Tokyo, Japan) as image capture software. Six sections per animal were selected, and Iba-1 and CD200 immunoreactive structure was evaluated by relative optical density (ROD) according to published methods [24,25,41] with modification. Namely, color images of the immunoreactive structure were converted to 8-bit greyscale images with a range of 0–255 (from black to white). The background level and the variance of the staining intensity were calculated on the 0–255 greyscale range. The percentile area of the significantly stained profiles and their average density in the area of interest were calculated using image analysis software from Image J (National Institutes of Health, Bethesda, MD, USA).

### 4.6. Double Immunofluorescence Staining

From the results of CD200 immunohistochemistry, we found that strong CD200 immunoreactivity was detected in neurons at 12 h after tFI and that CD200 immunoreactivity is primarily found in non-pyramidal cells in the CA1 field at 5 days after tFI. Therefore, to confirm the type of CD200 immunoreactive cells, double immunofluorescence staining was performed using goat anti-CD200 (diluted 1:50, R&D systems, MN, USA) with mouse anti-NeuN (diluted 1:1,100, Chemicon, Temecula, CA, USA) for neurons, rabbit anti-Iba-1 (diluted 1:400, Wako Chemicals, Richmond, VA, USA) for microglia or rabbit anti-GAD65/67 (diluted 1:200, Chemicon, Temecula, CA, USA) for GABAergic interneurons. The sections obtained at 12 h or 5 days post-tFI were incubated in the mixture of antisera for 8 h at 4 °C. They were briefly washed and reacted with a mixture of both Cy3-conjugated donkey anti-goat IgG (diluted 1:200; Jackson ImmunoResearch, West Grove, PA, USA) and FITC-conjugated donkey anti-rabbit IgG (diluted 1:200; Jackson ImmunoResearch West Grove, PA, USA) for 2 h at room temperature. The double immunoreaction was examined with confocal MS from LSM510 META NLO (Carl Zeiss, Oberkochen, Germany).

The numbers of Iba-1 immunoreactive microglia, GAD65/67 immunoreactive GABAergic interneurons, and NeuN immunoreactive pyramidal neurons, which were co-localized with CD200, were counted in a 400 μm^2^ square applied approximately at the center of the hippocampal CA1 field. The sections were selected with 120 μm intervals, and cell counts were obtained by averaging the counts from each gerbil.

### 4.7. Posttreatment with RIS

To investigate whether CD200 affected the neuroprotection of the CA1 pyramidal neurons from tFI injury, 10 mg/kg RIS was treated after tFI according to a published method [36]. It was reported that treatment with RIS, an atypical antipsychotic drug, after tFI induced hypothermia and provided neuroprotection in the gerbil hippocampal CA1 field by decreasing oxidative stress (Yang et al., 2019). In short, the gerbils (subtotal *n* = 40) were assigned to 4 groups: (1) sham/vehicle group (*n* = 5), which was given sham surgery for tFI and intraperitoneally injected with vehicle (0.3% Tween 80 in saline); (2) ischemia/vehicle group (*n* = 5), which was given tFI and intraperitoneally injected with vehicle; (3) sham/RIS group (*n* = 5), which was subjected to sham operation and intraperitoneally injected with RIS; and (4) ischemia/RIS group (*n* = 25), which was subjected to tFI and treated with RIS.

The gerbils were immediately treated with vehicle and/or RIS after tFI, underwent sham and/or tFI, and were sacrificed at 5 days post-tFI to investigate the neuroprotective effect of RIS against tFI. In addition, the gerbils belonging to the ischemia/RIS group were sacrificed at 6 h, 12 h, 1 day, 2 days, and 5 days to examine the change in CD200 immunoreactivity (Figure 9).

The brain section, immunohistochemistry for NeuN and CD200, and their analyses were performed using the same methods as described above.

### 4.8. Statistical Analysis

The data shown in this study represent the means ± SEM. The data were statistically analyzed by using SPSS 18.0 (SPSS, Chicago, IL, USA). Analysis of variance (ANOVA) with Bonferroni’s multiple comparison post hoc test was performed to determine differences among groups. *p* < 0.05 was used for statistical significance.

## Data Availability

The data presented in this study are available on request from the corresponding author.
